# Anatomical Evaluation of Localized Mandibular Trabecular Texture Differences in Children with Hypodontia Using Radiographic Fractal Analysis

**DOI:** 10.3390/diagnostics16162627

**Published:** 2026-08-19

**Authors:** Mert Nahir, Canan Bayraktar Nahir

**Affiliations:** 1 Department of Anatomy, Faculty of Medicine, Tokat Gaziosmanpaşa University, 60250 Tokat, Türkiye; mert.nahir@gmail.com; 2 Department of Pediatric Dentistry, Faculty of Dentistry, Tokat Gaziosmanpaşa University, 60250 Tokat, Türkiye

**Keywords:** congenital tooth agenesis, mandibular premolar, panoramic radiography, regions of interest, box-counting method, diagnostic imaging

## Abstract

**Background/Objectives:** This study aimed to evaluate mandibular trabecular bone structure in children with and without hypodontia using fractal analysis (FA) and to determine whether fractal dimension (FD) values vary according to hypodontia pattern and the localization of the affected alveolar region. **Methods:** This study included 75 children aged 8–12 years, divided into three age- and gender-matched groups: controls without agenesis (Group 1, *n* = 25), mandibular second premolar agenesis (Group 2, *n* = 25), and agenesis of both mandibular first and second premolars (Group 3, *n* = 25). Panoramic radiographs were analyzed using a semi-automated FA method involving manual ROI placement followed by automated image processing and FD calculation. FD values were measured in three regions of interest (ROIs): the mandibular condyle (ROI1), mandibular angle (ROI2), and alveolar region corresponding to the agenetic area (ROI3). **Results:** FD values did not differ significantly by age or gender in any ROI (*p* > 0.05). The pattern of FD differences among the study groups varied significantly according to anatomical region, as demonstrated by the significant Group × ROI interaction (*p* < 0.001). Post hoc comparisons showed that the most pronounced intergroup difference occurred in ROI3, where Group 2 had a significantly lower FD value (0.78 ± 0.26) than Group 1 (1.10 ± 0.21) and Group 3 (1.04 ± 0.23). **Conclusions:** Mandibular trabecular texture differences in children with hypodontia appeared to be localized mainly to the alveolar region corresponding to tooth agenesis. The lower FD value observed in the mandibular second premolar agenesis group suggests that these regional differences may be related to the anatomical localization of the agenetic area rather than to the number of missing teeth alone. Although such findings may help draw attention to site-specific alveolar changes during radiographic assessment, they are based on two-dimensional panoramic radiographic texture analysis and should be regarded as preliminary and hypothesis-generating.

## 1. Introduction

Congenital tooth agenesis is defined as the absence of one or more teeth due to developmental failure and is considered one of the most common dental anomalies [1]. The congenital absence of one to five teeth, excluding third molars, is termed hypodontia; the absence of six or more teeth is referred to as oligodontia; and the complete absence of all teeth is defined as anodontia [2]. Tooth agenesis is frequently associated with impaired masticatory function, speech difficulties, and significant aesthetic concerns, all of which may adversely affect the psychological well-being of the developing child [3]. However, its consequences are not limited to these clinical manifestations, as the potential impact of tooth agenesis on the quality of the underlying mandibular bone is also of considerable clinical importance.

Alveolar bone is a dynamic tissue whose growth and remodeling are strongly influenced by the presence of teeth and the functional loading generated during mastication. The absence of a tooth germ may disrupt normal alveolar process development and result in localized alterations in bone structure and morphology [4]. Previous studies have similarly associated tooth agenesis with reduced alveolar development and changes in mandibular trabecular architecture [5,6]. Detecting such site-specific alterations may be clinically relevant in children with hypodontia, as the affected regions may require closer evaluation during growth and before orthodontic space management, prosthetic rehabilitation, or future implant-related treatment [7]. Although fractal analysis is not currently suitable as a stand-alone diagnostic or treatment-planning tool, it may provide complementary information on regional radiographic trabecular texture and support a more individualized assessment of alveolar development.

Traditionally, bone quality has been assessed using radiomorphometric indices such as the Mandibular Cortical Index (MCI) and the Panoramic Mandibular Index (PMI), both of which primarily rely on cortical bone evaluation [8]. However, trabecular bone is metabolically more active and is considered more sensitive to early structural changes. For this reason, fractal analysis (FA) has recently become a widely used method in dental radiology for the quantitative assessment of trabecular bone architecture [9]. Through fractal dimension (FD) calculation, the complexity of the radiographic trabecular pattern can be expressed numerically; higher FD values generally indicate a more complex radiographic texture, whereas lower values reflect a simpler pattern. Accordingly, FA may be useful for identifying subtle differences in radiographic trabecular texture that may not be readily apparent on visual assessment [10].

FA has been widely applied in dental and medical research to investigate bone alterations associated with osteoporosis, periodontitis, implant stability, and various systemic conditions [11,12]. Despite its increasing use, studies evaluating trabecular bone structure in patients with tooth agenesis remain limited [7,13,14]. Although recent studies have evaluated mandibular trabecular bone structure in patients with mandibular second premolar hypodontia, the available evidence has largely focused on single-tooth agenesis patterns [7,14]. It therefore remains uncertain whether the observed trabecular differences are confined to the anatomical region of agenesis, reflect a more generalized mandibular alteration, or vary according to the pattern and extent of tooth absence. In particular, comparative data on isolated mandibular second premolar agenesis and combined agenesis of the mandibular first and second premolars are limited. Evaluating these distinct hypodontia patterns together with anatomically distant mandibular regions may help clarify whether radiographic trabecular texture differences are more closely associated with the localization of the agenetic region than with the number of missing teeth alone. The study was restricted to mandibular premolar agenesis because these patterns provide anatomically comparable posterior mandibular regions for ROI placement while reducing heterogeneity related to tooth type and jaw location [7,14]. This focused approach therefore allows a more direct evaluation of whether trabecular differences are associated primarily with the localization or extent of agenesis.

This study aimed to evaluate the mandibular radiographic trabecular texture in children with and without tooth agenesis using FA and to determine whether FD values vary according to hypodontia pattern and the localization of the affected alveolar region. The null hypothesis was that the mandibular trabecular bone structure, as assessed by FA, would not differ significantly among children without tooth agenesis and those with different hypodontia patterns involving mandibular premolar agenesis.

## 2. Materials and Methods

### 2.1. Ethical Statement

This retrospective cross-sectional study was approved by the Non-Interventional Scientific Research Ethics Committee of Tokat Gaziosmanpaşa University (Approval No. 26-MOBAEK-029; 3 February 2026). The study was conducted in accordance with the principles of the Declaration of Helsinki and reported in compliance with the Strengthening the Reporting of Observational Studies in Epidemiology (STROBE) guidelines.

### 2.2. Sample Size Calculation

The required sample size was calculated using G*Power software (version 3.1.9.2; Franz Faul, Kiel University, Germany). The effect size was derived from a previous panoramic radiographic study that used fractal analysis to compare mandibular trabecular bone among independent groups [15]. Although the reference population did not specifically consist of patients with hypodontia, the study was selected because of its methodological similarity in terms of imaging modality, FD-based assessment, and group-comparison design. Based on an effect size of f = 0.47, a significance level of 0.05, and a statistical power of 95%, the minimum required sample size was calculated using the “ANOVA: Fixed effects, omnibus, one-way” option. Accordingly, a total of 75 participants was required.

### 2.3. Patient and Radiographic Image Selection

This retrospective study evaluated the radiographic records of pediatric patients aged 8–12 years who presented to the Department of Pediatric Dentistry, Faculty of Dentistry, Tokat Gaziosmanpaşa University, Tokat, Türkiye, for routine dental examination and treatment between 1 January 2021 and 1 January 2026. Written informed consent for the use of clinical and radiographic data for research purposes was obtained from the participants’ parents or legal guardians.

Congenital tooth agenesis was confirmed through a combined review of panoramic radiographs, clinical records, and dental history. Agenesis was diagnosed when no mineralized tooth germ was visible in the expected anatomical region, and there was no documented history or radiographic evidence of previous extraction, trauma, or surgical removal. To reduce the possibility of misclassifying delayed dental development as agenesis, only patients within the predefined age range whose overall dental development was compatible with the expected stage were included. The presence of the corresponding retained primary tooth or teeth was also verified clinically and radiographically.

Inclusion and exclusion criteria were established a priori for all study groups. Eligible participants included individuals with congenital tooth agenesis of the mandibular second premolar or of both the mandibular first and second premolars. Retention of the corresponding primary teeth, specifically the primary second molar or the primary first and second molars, was also required. To reduce the potential effects of eruption-related physiological changes on alveolar bone and occlusal pattern, only individuals with completed root development and erupted permanent first molars were included. To standardize ROI placement and laterality, only patients with unilateral left-sided mandibular premolar agenesis were included in the hypodontia groups. Patients with isolated right-sided agenesis and those with bilateral agenesis were excluded, and no radiographs were mirrored.

Patients were excluded when carious lesions, recurrent caries, or associated apical or periodontal pathology were present within or immediately adjacent to the predefined ROI and could affect radiographic trabecular texture. Caries located outside the measurement region did not constitute an exclusion criterion. Patients with restorations involving the reference teeth or extending into the ROI were excluded. Additional exclusion criteria included systemic diseases or medications affecting bone metabolism and a history of syndromes or genetic disorders.

To ensure diagnostic reliability in radiographic assessment, images with compromised diagnostic quality resulting from patient movement, positioning errors, or other technical artifacts were excluded. All panoramic radiographs were graded according to the criteria of the UK National Radiological Protection Board [16]. Only Grade 1 radiographs, defined as diagnostically perfect images without exposure, positioning, or processing errors, were included in the study.

The study sample consisted of children in the mixed dentition period. Eligible participants were assigned to three groups according to tooth agenesis status: Group 1 comprised children without tooth agenesis, Group 2 comprised children with unilateral left-sided mandibular second premolar agenesis, and Group 3 comprised children with unilateral left-sided agenesis of both the mandibular first and second premolars. After the eligible hypodontia cases had been identified, 25 control participants were selected from the larger pool of eligible children without tooth agenesis. The control group was frequency-matched to the hypodontia groups according to age and sex distribution to improve comparability across groups.

All panoramic radiographs were obtained at the Department of Oral and Maxillofacial Radiology, Faculty of Dentistry, Tokat Gaziosmanpaşa University. The images were acquired by the same technician using a single panoramic unit (Soredex Cranex Novus, Tuusula, Finland) under standardized exposure parameters of approximately 70 kVp, 10 mA, and an exposure time of 8 s. The panoramic radiographs were retrieved from the institutional image database and exported using Planmeca Romexis 3.8.3 software (Helsinki, Finland) in high-resolution Joint Photographic Experts Group (JPEG) format with an 8-bit depth. Identical export settings were applied to all images, and no additional resizing, enhancement, or format conversion was performed before fractal analysis. The exact physical pixel size and spatial resolution were not available in the retrospectively archived JPEG metadata.

### 2.4. Fractal Dimension Analysis

FA was performed using the “*single-click automated fractal analysis*” module developed by Balel and Sağtaş [17]. The module was implemented in Python (version 3.11) using open-source libraries, including OpenCV (cv2; version 4.11.0), NumPy (version 2.2.4), and scikit-image (version 0.26.0), and is publicly available at GitHub (https://github.com/semruktech/fractal-box-counter) (accessed on 5 March 2026). In the present study, the ROI size was predefined as 20 × 20 pixels, and the examiner manually selected the center of each ROI according to the anatomical landmarks described below. Once the ROI was selected, the subsequent processing and FD calculation were performed automatically by the module. Briefly, the selected image region was subjected to Gaussian filtering to reduce large-scale variations in image brightness. The filtered image was then processed relative to the original ROI, followed by gray-level adjustment and conversion to a binary image. Morphological operations were subsequently applied to refine the trabecular pattern, after which the image was skeletonized to obtain a one-pixel-wide representation of the trabecular structure. Finally, the fractal dimension was calculated automatically using the box-counting method. Thus, manual interaction was limited to anatomical ROI placement, whereas image processing and FD calculation were performed through a standardized automated workflow [17].

**Figure 1:**
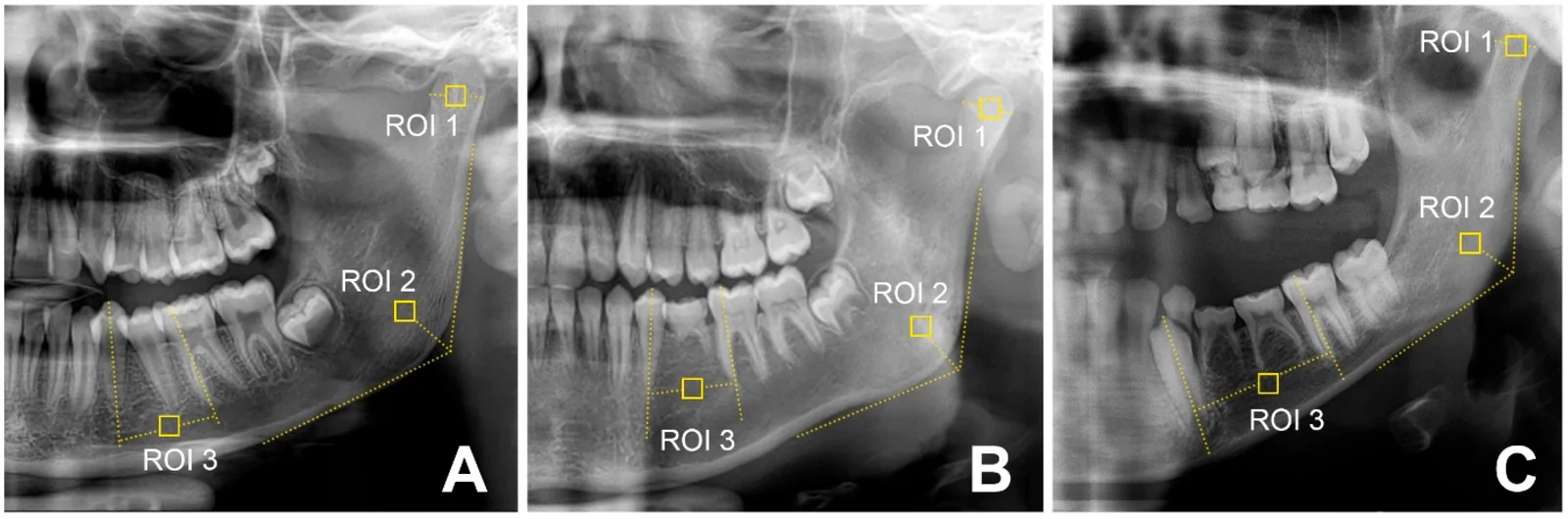
Manual selection of the ROIs for FA on panoramic radiographs. ROI1 was placed at the center of the left mandibular condyle, ROI2 at the center of the left mandibular angle, and ROI3 in the alveolar region corresponding to the agenetic tooth/teeth. Representative examples are shown in panels (**A**–**C**): (**A**) Group 1; (**B**) Group 2; (**C**) Group 3.

The first step of the analysis consisted of manual ROI selection based on the predefined anatomical landmarks described above. After ROI placement, all subsequent image-processing steps, including grayscale normalization, filtering, binarization, morphological operations, skeletonization, and fractal box-counting, were performed automatically by the software. Thus, the automated workflow reduced user-dependent variability during image processing and FD calculation, although potential observer variability related to manual ROI placement remained. ROIs were selected from the following anatomical areas in the left posterior mandible (Figure 1).

ROI1: Center of the left mandibular condyle.
ROI2: Center of the left mandibular angle.
ROI3: ROI3 was placed in the left posterior alveolar region corresponding to the tooth or teeth absent in the hypodontia groups.

In Group 1, the ROI was positioned in the mandibular second premolar region, midway between reference lines drawn through the root canal of the first premolar and the mesial root canal of the first molar. The same landmarks were used in Group 2, in which the mandibular second premolar was agenetic. In Group 3, because both mandibular premolars were agenetic, the ROI was positioned midway between reference lines drawn through the root canal of the canine and the mesial root canal of the first molar. In all groups, ROI3 was placed at the apical level of the reference teeth on the left side of the mandible, and a constant ROI size of 20 × 20 pixels was used. Thus, comparability was supported by standardizing the mandibular side, vertical level, ROI size, and landmark-based midpoint placement. However, because the anatomical extent of the agenetic region differed between Groups 2 and 3, exact spatial equivalence of ROI3 across all groups could not be achieved.

Because no universally accepted ROI size has been established for fractal analysis in panoramic radiographs, a fixed 20 × 20-pixel ROI was chosen according to the anatomical constraints of the pediatric alveolar region and the requirements of the automated workflow. Its relatively small size permitted placement within the pediatric alveolar region while minimizing the inclusion of adjacent dental roots, periodontal structures, cortical boundaries, and other anatomical features. This approach was further supported by the use of the same panoramic unit, standardized acquisition parameters, and identical image-export settings for all radiographs. Nevertheless, a fixed pixel-based ROI did not necessarily represent an identical physical anatomical area in every participant.

A standardized ROI based on a stable mandibular landmark, such as the inferior mandibular cortex, was considered but not adopted because the primary objective was to evaluate the alveolar region most closely corresponding to the site of tooth agenesis rather than a fixed basal mandibular region. During mixed dentition, the vertical relationship between the alveolar process and the inferior mandibular cortex may vary with growth, eruption stage, and local alveolar development. Consequently, a fixed cortical reference could have displaced the ROI from the alveolar region most relevant to the agenetic site. Nevertheless, the use of group-specific dental landmarks introduced a potential source of measurement bias because ROI3 did not represent an identical anatomical location in all groups.

### 2.5. Reliability of Measurements

All ROIs were manually placed by a single anatomist (M.N.) using the predefined anatomical landmarks. Before analysis, all radiographs were anonymized and coded. The examiner was blinded to patient identifiers, age, sex, and clinical record information during ROI placement. Blinding to hypodontia group allocation was not possible because the presence and anatomical pattern of tooth agenesis were directly visible on the panoramic radiographs. After ROI placement, image processing and FD calculation were performed automatically by the software. To assess intra-observer reliability, all three ROI measurements (ROI1, ROI2, and ROI3) were repeated in 20 randomly selected participants two weeks after the initial assessment. Intra-observer reliability was evaluated using a two-way mixed-effects model with absolute agreement for single measurements [ICC(3,1)].

### 2.6. Statistical Analysis

The data were analyzed using IBM SPSS Statistics for Windows version 26.0 (SPSS Inc., Chicago, IL, USA). Descriptive statistics for quantitative variables are presented as mean ± standard deviation, while categorical variables are summarized as frequency (percentage). Normality of quantitative data was assessed using the Kolmogorov–Smirnov test, which was selected considering the total sample size (*n* = 75) of the study. An independent-sample *t*-test was used for comparisons between two independent groups, while one-way analysis of variance (ANOVA) was applied for comparisons among three independent groups. A mixed-design repeated-measures analysis of variance (ANOVA) was performed to evaluate the effects of study group and anatomical region on FD values. Study group (Group 1, Group 2, and Group 3) was included as the between-subject factor, whereas ROI (ROI1, ROI2, and ROI3) was treated as the within-subject repeated factor. The main effects of group and ROI, as well as the Group × ROI interaction, were examined. The assumption of sphericity was assessed using Mauchly’s test. When the sphericity assumption was violated, Greenhouse–Geisser-corrected results were planned to be reported. Pairwise comparisons following significant main or interaction effects were performed using Bonferroni adjustment for multiple comparisons. Effect sizes were reported as partial eta squared (partial η^2^). Intraobserver reliability was determined by calculating the intraclass correlation coefficient (ICC). Reliability was interpreted as follows: values < 0.50 were considered poor, 0.50–0.75 moderate, 0.75–0.90 good, and >0.90 excellent. Statistical significance was set at *p* < 0.05.

## 3. Results

Intra-observer reliability was assessed using a two-way mixed-effects, absolute-agreement, single-measure ICC [ICC(3,1)]. The ICC values for ROI1, ROI2, and ROI3 were 0.870, 0.958, and 0.883, respectively, indicating good to excellent intra-observer agreement.

A total of 229 panoramic radiographs were screened during the retrospective review. Cases with isolated right-sided or bilateral mandibular premolar agenesis were not included in the 229 radiographs screened for the final eligibility assessment because laterality was applied as a prespecified sampling criterion. Among these, 46 were excluded because of poor image quality related to patient movement, positioning errors, or technical artifacts. Additional exclusions were made for dental caries in the region of interest (*n* = 32), absence of retained primary teeth in the agenetic region (*n* = 23), apical or periodontal pathology in the measurement area (*n* = 10), systemic diseases or medication use affecting bone metabolism (*n* = 5), a history of syndromes or genetic disorders (*n* = 7), and previous or ongoing orthodontic treatment (*n* = 16). Each excluded patient was assigned to a single primary exclusion category; therefore, the exclusion categories were mutually exclusive. Following application of the predefined inclusion and exclusion criteria, 90 children remained eligible for further evaluation, including 40 children without tooth agenesis, 25 children with unilateral left-sided mandibular second premolar agenesis, and 25 children with unilateral left-sided agenesis of both the mandibular first and second premolars. All eligible children in the two hypodontia groups were included in the study. Because the number of eligible controls exceeded the required sample size, 25 control participants were selected from the pool of 40 eligible children using frequency matching to achieve age and sex distributions comparable to those of the hypodontia groups. The final study sample therefore consisted of 75 children, with 25 participants in each group.

As the groups were matched for age and gender, each group consisted of 48% female and 52% male participants. The comparable age and sex distributions across the three groups reflect the frequency-matching procedure and were intentionally achieved during control selection. The mean ages of Groups 1, 2, and 3 were 10.40 ± 1.57, 10.45 ± 1.42, and 10.46 ± 1.50 years, respectively. No statistically significant differences were observed among the groups with respect to age or gender (*p* > 0.05). The distribution of the 75 participants by group, age, and gender is shown in Table 1.

The distribution of FD values across the ROIs according to gender and age is presented in Table 2. In the gender-based comparisons, no statistically significant differences were observed between female and male participants in ROI1, ROI2, and ROI3 (*p* = 0.677, *p* = 0.852, and *p* = 0.527, respectively). Similarly, comparisons across age groups showed no statistically significant differences in FD values for ROI1, ROI2, or ROI3 (*p* = 0.441, *p* = 0.071, and *p* = 0.818, respectively).

The mixed-design repeated-measures ANOVA revealed a significant main effect of group (F = 8.419, *p* = 0.001, partial η^2^ = 0.190) and a significant main effect of ROI (F = 4.117, *p* = 0.018, partial η^2^ = 0.054). Importantly, the Group × ROI interaction was also statistically significant (F = 5.483, *p* < 0.001, partial η^2^ = 0.132), indicating that the pattern of FD differences among the study groups varied according to anatomical region (Table 3).

The descriptive statistics and post hoc comparisons are presented in Table 4. Group 1 had an overall mean FD value of 1.09 ± 0.20, whereas Group 2 showed the lowest overall mean FD value at 0.95 ± 0.24, and Group 3 had an overall mean FD value of 1.04 ± 0.20. Across the anatomical regions, the mean FD values were 1.04 ± 0.18 for ROI1, 1.06 ± 0.19 for ROI2, and 0.97 ± 0.27 for ROI3. The most pronounced intergroup difference was observed in ROI3, where Group 2 had a significantly lower mean FD value (0.78 ± 0.26) than Group 1 (1.10 ± 0.21) and Group 3 (1.04 ± 0.23). This pattern was consistent with the significant Group × ROI interaction demonstrated in Table 3. Detailed post hoc pairwise comparisons, including mean differences, 95% confidence intervals, and Bonferroni-adjusted *p* values, are provided in the Supplementary Materials Tables S1–S3.

## 4. Discussion

In this study, the null hypothesis that mandibular trabecular bone structure, as assessed by FA, would not differ among children without tooth agenesis and those with different mandibular premolar hypodontia patterns was partially rejected. The findings indicated that radiographic trabecular texture differences were not observed throughout the mandible but were mainly identified in the alveolar region corresponding to tooth agenesis. Importantly, the significant Group × ROI interaction obtained from the mixed-design repeated-measures analysis demonstrated that the pattern of intergroup differences in FD varied according to anatomical region, while accounting for the repeated ROI measurements obtained from the same participants. Alveolar bone development is closely related to tooth presence. In addition, tooth development and craniofacial development share certain genetic and molecular mechanisms. These relationships raise the question of whether the bone changes observed in hypodontia are limited to the region of tooth absence [18]. Therefore, evaluation of the mandibular trabecular structure in children with hypodontia is important not only for characterizing tooth agenesis but also for understanding the biological response of the affected region.

In exploring this biological relationship, FA appears to be a suitable method for the non-invasive assessment of radiographic trabecular texture. The use of the box-counting method, which is widely employed for FD calculation, enhances the comparability of the findings with the existing literature [19]. Digital panoramic radiography was considered appropriate for this pediatric sample. It is widely used in routine clinical practice, covers a broad anatomical area, and involves a lower radiation dose than cone-beam computed tomography (CBCT) [20]. However, FD values derived from panoramic radiographs should be interpreted as two-dimensional radiographic texture characteristics rather than direct measurements of true three-dimensional trabecular microarchitecture. Therefore, while FA can provide useful information about radiographic trabecular complexity, it cannot fully represent the spatial organization, thickness, connectivity, or volumetric properties of trabecular bone. In addition, FA is not yet integrated into routine clinical workflows and generally requires dedicated image processing or post-processing procedures. For this reason, its translational relevance should currently be considered preliminary rather than directly clinical [21]. Given that FD may also be influenced by ROI selection, shape, and placement, the use of planar ROIs and a standardized measurement protocol supports methodological consistency [22]. Although anatomical limitations related to the mixed dentition period and variation in reference regions according to the hypodontia pattern required some adaptations in ROI placement, repeat measurements together with high intra-observer agreement supported the reliability of the measurement protocol.

Within this methodological framework, FD values did not differ significantly according to age or gender in ROI1, ROI2, or ROI3. This finding is consistent with the limited literature available on studies involving the pediatric population [7,14]. Temur et al. [7] reported that, in children and adolescents with mandibular second premolar hypodontia, FD values were not influenced by age or gender, whereas the observed difference was localized to the region corresponding to the agenetic tooth. Similarly, Gökkurt Yılmaz et al. [14] found that FD values in individuals with hypodontia did not differ between genders and were not significantly associated with age. In the present study, FD values did not differ significantly according to age or gender in any ROI. This suggests that mandibular trabecular structure during childhood may be influenced more by the local dentoalveolar environment than by demographic variables. Biomechanical stimuli in the region of tooth agenesis and alveolar developmental dynamics may also contribute to this pattern. In addition, the relatively homogeneous growth period represented by the 8–12-year age range may explain why age- and gender-related differences were not apparent.

When all groups were considered together, the significant differences observed in FD values among the ROIs suggest that mandibular trabecular bone does not exhibit a regionally homogeneous structure. In the present study, the highest mean FD value was observed in ROI2 (1.06 ± 0.19), whereas the lowest was found in ROI3 (0.97 ± 0.27); the mean FD value of ROI1 (1.04 ± 0.18) was comparable to those of both regions. This distribution suggests that the alveolar region may have lower trabecular complexity than other mandibular areas. It may also be more directly influenced by tooth presence and functional loading. Consistent with this interpretation, previous studies have reported lower FD values in the alveolar ROI corresponding to the region of tooth agenesis, whereas no significant changes were generally observed in more distant anatomical regions such as the ramus and condyle in cases of single-tooth hypodontia [7,14]. In addition, regional differences in the direction and magnitude of occlusal forces have been reported to influence trabecular bone organization, which may contribute to lower FD values in alveolar areas adjacent to teeth [23]. Therefore, ROI3 may represent a region in which radiographic trabecular texture differences associated with tooth agenesis are more readily observed.

The significant Group × ROI interaction indicates that the effect of study group on FD was dependent on anatomical region. Post hoc comparisons further showed that the most pronounced intergroup difference occurred in ROI3, where Group 2 exhibited lower FD values than Groups 1 and 3, whereas comparable differences were not observed in ROI1 or ROI2. This interaction provides statistical support for the interpretation that the observed group differences were region-dependent rather than uniformly distributed throughout the mandible. This interpretation is consistent with the findings of Temur et al. [7], who reported no significant differences in ROI1 and ROI2 in children and adolescents with mandibular second premolar hypodontia but lower FD values in ROI3, which represented the agenetic region. Similarly, a more recent study demonstrated a marked reduction in FD, particularly at the ROI3 level, in the hypodontia group, and attributed this finding to the inability of the retained primary second molar to fully preserve normal trabecular maturation in the absence of the permanent second premolar [14]. In addition, Ostler et al. [24] reported a progressive reduction in alveolar ridge width in the absence of the mandibular second premolar, whereas Bertl et al. [4] showed that premolar agenesis was associated with dimensional and morphological changes in mandibular form. Together, these findings suggest that the ROI3-related result may reflect a localized radiographic trabecular alteration. However, because the analysis was based on two-dimensional panoramic radiographs, this finding should not be interpreted as a direct measurement of three-dimensional bone morphology. In this context, the low FD values observed in ROI3 may be associated with tooth agenesis, insufficient alveolar development, reduced physiological occlusal loading, and incomplete trabecular maturation in a region lacking eruption potential.

Although localized trabecular changes associated with hypodontia have been reported, the present findings suggest that these alterations should not be interpreted simply as a linear effect of the number of agenetic teeth. Créton et al. [13] reported changes in certain trabecular parameters and in fractal dimension in relation to hypodontia, although a direct distinction between hypodontia and control groups was not consistently demonstrated. Such variability may be related to differences in ROI selection, defect localization, sample characteristics, and compensatory structural adaptation in the surrounding bone. In the present study, the lowest FD value in ROI3 was observed in Group 2 rather than in Group 3. Therefore, this finding should be interpreted primarily as a localized alveolar alteration associated with the anatomical region corresponding to mandibular second premolar agenesis, rather than as evidence of a direct relationship between FD reduction and the number of missing teeth. The ROI3 value in Group 2 was 0.78 ± 0.26, compared with 1.10 ± 0.21 in the control group and 1.04 ± 0.23 in Group 3, and multiple comparison analysis confirmed that the significant difference originated from Group 2. This more pronounced reduction may be explained by the fact that ROI3 in Group 2 directly represented the alveolar area where the mandibular second premolar would normally develop, a region directly affected by the absence of the tooth germ, reduced eruptive stimulus, and altered occlusal force transmission. In contrast, the wider definition of ROI3 in Group 3, extending between the canine and first molar, may have partially diluted or obscured focal trabecular alterations. Thus, the ROI3 comparison should be interpreted not only as a comparison between hypodontia groups but also in light of the anatomical extent and localization represented by the selected ROI. Taken together, these findings emphasize that trabecular changes associated with hypodontia should be interpreted in relation to the localization of the agenetic region, the biological representativeness of the selected ROI, and regional functional loading conditions, rather than the number of agenetic teeth alone. Nevertheless, these interpretations should be regarded as biologically plausible hypotheses, as the present study did not directly assess alveolar development, eruptive stimuli, occlusal loading, or three-dimensional trabecular architecture.

From a clinical perspective, the localized FD differences observed in ROI3 may draw attention to alveolar regions that warrant closer evaluation during the long-term management of children with hypodontia. In patients for whom orthodontic space closure, maintenance of retained primary teeth, prosthetic rehabilitation, or future implant therapy is being considered, a lower FD value may indicate altered radiographic trabecular texture and may complement conventional anatomical and clinical assessment. In particular, when future implant rehabilitation is planned, such regions may merit additional evaluation of alveolar ridge width, height, and three-dimensional morphology using appropriate imaging when clinically justified. However, these findings should currently be regarded as hypothesis-generating rather than clinically actionable. The present study does not establish clinically meaningful FD thresholds for diagnosis or treatment planning and does not demonstrate that FD can predict the need for bone augmentation or grafting. Therefore, the potential value of FD as a supplementary marker for treatment planning should be validated prospectively against three-dimensional alveolar measurements and actual treatment requirements.

One of the major strengths of this study is that it evaluated mandibular trabecular bone changes associated with hypodontia in a pediatric population and compared cases according to the number of agenetic teeth. The use of age- and gender-matched groups, a homogeneous sample, and strict exclusion of potential confounding factors affecting trabecular structure strengthened the interpretability of the findings. In addition, as one of the few studies to demonstrate differences in mandibular radiographic trabecular texture in children and adolescents with hypodontia, this study provides an important foundation for future research.

However, the retrospective cross-sectional design does not allow assessment of the temporal progression of the observed trabecular changes or the establishment of causal relationships. The findings are limited to the 8–12-year age window represented in this study and may not reflect trabecular texture characteristics at earlier or later stages of craniofacial growth, dental eruption, and alveolar development. In addition, the data were obtained from a single center and were based solely on two-dimensional panoramic radiographs. The retrospective, single-center sampling process may also have introduced selection bias. The study included only children who attended a tertiary dental clinic, had diagnostically acceptable panoramic radiographs, and met the strict eligibility criteria; therefore, the final sample may not fully represent the broader population of children with hypodontia. In particular, the exclusion of patients with right-sided agenesis, poor-quality images, orthodontic treatment history, or coexisting dental and systemic conditions may have resulted in a more homogeneous but selectively defined cohort. These factors limit the generalizability of the findings, while the use of two-dimensional panoramic radiographs also restricts the direct evaluation of true three-dimensional bone microarchitecture. Therefore, FD values derived from panoramic radiographs should be interpreted as indicators of radiographic trabecular texture rather than as direct representations of the spatial organization, thickness, connectivity, or volumetric properties of trabecular bone. A major methodological limitation of this study is that ROI3 was defined using different anatomical landmarks across the study groups according to the location and extent of agenesis. In the present study, ROI3 was defined using group-specific anatomical reference teeth in order to represent the alveolar region corresponding to the agenetic tooth/teeth as accurately as possible. Although this approach was clinically justified, it may have introduced some variability in anatomical comparability between groups. The mandibular side, ROI size, vertical level, and landmark-based midpoint placement were standardized; exact anatomical equivalence could not be achieved because ROI3 represented different extents of the alveolar region in the isolated and combined agenesis groups. In addition, the use of a fixed 20 × 20-pixel ROI standardized the amount of image data analyzed but did not guarantee an identical physical anatomical area across patients, because panoramic magnification and patient dimensions may vary. ROI size itself may also influence FD measurements, as previous studies have shown that differences in ROI size, shape, and localization can affect calculated fractal dimensions [22]. In particular, relatively small ROIs may be more susceptible to local sampling variability and ROI-selection bias; therefore, the use of a 20 × 20-pixel ROI should be considered an additional methodological limitation of the present study. Therefore, part of the observed intergroup difference may reflect regional variation in radiographic trabecular texture in addition to the effect of tooth agenesis. This group-dependent ROI definition represents a potential measurement bias and is particularly relevant to the comparison between Groups 2 and 3. Accordingly, the lower FD value observed in Group 2 cannot be attributed exclusively to the biological effect of hypodontia, because differences in the anatomical location and extent of ROI3 may also have contributed to the result. Because trabecular bone structure can vary according to anatomical location, local morphology, and functional loading, ROI3-related findings should be interpreted with caution. This consideration may partly explain why the lowest FD value was observed in Group 2 rather than in Group 3, despite the greater number of agenetic teeth in Group 3. Another limitation is that alveolar ridge dimensions, primary tooth exfoliation timing, and detailed occlusal relationships were not assessed. Consequently, the relationship between the observed radiographic trabecular texture differences and these clinically relevant developmental parameters remains unknown, limiting the immediate clinical applicability of the findings. Furthermore, because this study intentionally excluded children with systemic conditions or medication use affecting bone metabolism, the findings should be interpreted within the context of otherwise healthy pediatric patients with localized tooth agenesis. Accordingly, the results cannot be extrapolated to populations with systemic skeletal alterations or broader variations in bone metabolism. This design allowed the local dentoalveolar effect of hypodontia to be evaluated with fewer systemic confounding factors; however, it also limits conclusions regarding generalized mandibular or skeletal trabecular changes.

Future research should primarily focus on prospective validation of these findings in larger, multicenter pediatric cohorts and on correlating panoramic FD measurements with three-dimensional imaging parameters. Where clinically justified, low-dose CBCT protocols may provide additional information on alveolar ridge dimensions and three-dimensional trabecular architecture, allowing comparison with two-dimensional FD measurements while minimizing radiation exposure in pediatric patients. Such studies would help determine whether the observed two-dimensional radiographic texture differences correspond to true volumetric trabecular alterations and whether they have value for longitudinal assessment during growth. Subsequent investigations may also evaluate different hypodontia patterns, systemic variations in bone metabolism, and ROI protocols based on stable mandibular landmarks to improve anatomical comparability and methodological reproducibility.

## 5. Conclusions

This study showed that radiographic trabecular texture differences in children with hypodontia were not generalized throughout the mandible but were mainly localized to the alveolar region corresponding to tooth agenesis. The lower FD value observed in ROI3, particularly in the mandibular second premolar agenesis group, suggests that these regional differences may be influenced by the anatomical localization of the agenetic area rather than by the number of missing teeth alone. However, because the findings are based on two-dimensional panoramic radiographic texture analysis and group-specific ROI definitions, they should not be interpreted as direct evidence of altered three-dimensional bone microarchitecture or as a basis for immediate clinical decision-making. At present, FD assessment may be regarded primarily as a research approach for characterizing localized alveolar differences and generating hypotheses for prospective, longitudinal, and three-dimensional validation studies. Its potential role in the assessment or management of children with hypodontia remains to be established.

## Figures and Tables

**Table 1:** Distribution of children according to group, age, and gender.

Age	Group 1		Group 2		Group 3		Total *n* (%)
	Female *n* (%)	Male *n* (%)	Female *n* (%)	Male *n* (%)	Female *n* (%)	Male *n* (%)	
8–8.99	2 (8)	3 (12)	2 (8)	3 (12)	2 (8)	3 (12)	15 (20)
9–9.99	2 (8)	3 (12)	2 (8)	3 (12)	2 (8)	3 (12)	15 (20)
10–10.99	2 (8)	3 (12)	2 (8)	3 (12)	2 (8)	3 (12)	15 (20)
11–11.99	3 (12)	2 (8)	3 (12)	2 (8)	3 (12)	2 (8)	15 (20)
12–12.99	3 (12)	2 (8)	3 (12)	2 (8)	3 (12)	2 (8)	15 (20)
Total	25 (33.3)		25 (33.3)		25 (33.3)		75 (100)

*n* (%): number of children (percentage value).

**Table 2:** Comparison of FD values of ROIs by gender and age.

	ROI1	*p*	ROI2	*p*	ROI3	*p*
Gender						
Female	1.04 ± 0.17	0.677 *	1.06 ± 0.15	0.852 *	0.95 ± 0.20	0.527 *
Male	1.05 ± 0.19		1.06 ± 0.21		0.99 ± 0.32	
Age						
8–8.99	1.02 ± 0.21	0.441 **	1.09 ± 0.13	0.071 **	1.04 ± 0.25	0.818 **
9–9.99	1.04 ± 0.13		1.02 ± 0.27		0.96 ± 0.26	
10–10.99	1.01 ± 0.23		1.02 ± 0.15		0.99 ± 0.30	
11–11.99	1.12 ± 0.15		1.00 ± 0.17		0.92 ± 0.29	
12–12.99	1.03 ± 0.16		1.17 ± 0.14		0.96 ± 0.26	

The data are presented as the mean ± standard deviation (SD) for continuous data. ROI1: center of the left mandibular condyle; ROI2: center of the left mandibular angle; ROI3: left alveolar region of tooth agenesis. Statistically significant at *p*-value < 0.05. * Independent-sample *t*-test. ** One-way analysis of variance (ANOVA).

**Table 3:** Evaluation of the effects of group and ROIs on FD.

	Type III Sum of Squares	df	Mean Square	F	*p*	Partial Eta Squared
Group	0.720	2	0.360	8.419	**0.001**	0.190
ROIs	0.322	2	0.161	4.117	**0.018**	0.054
Group × ROIs	0.859	4	0.215	5.483	**<0.001**	0.132

Abbreviations: df: degrees of freedom; F: mixed-design repeated-measures ANOVA test statistic. Statistically significant at *p*-value < 0.05.

**Table 4:** Comparison of FD values of ROIs among groups.

Group	ROIs			Total
	ROI1	ROI2	ROI3	
Group 1	1.11 ± 0.20 ^x^	1.05 ± 0.18 ^x^	1.10 ± 0.21 ^x^	1.09 ± 0.20 ^a^
Group 2	1.02 ± 0.14 ^x^	1.05 ± 0.22 ^x^	0.78 ± 0.26 ^y^	0.95 ± 0.24 ^b^
Group 3	1.00 ± 0.19 ^x^	1.09 ± 0.16 ^x^	1.04 ± 0.23 ^x^	1.04 ± 0.20 ^a^
Total	1.04 ± 0.18 ^AB^	1.06 ± 0.19 ^B^	0.97 ± 0.27 ^A^	1.03 ± 0.22

Data are presented as mean ± standard deviation. ROI1: center of the left mandibular condyle; ROI2: center of the left mandibular angle; ROI3: left alveolar region of tooth agenesis. Lowercase letters (^a,b^) indicate post hoc comparisons among the study groups based on overall FD values. Groups sharing the same lowercase letter are not significantly different, whereas groups with different lowercase letters differ significantly. Uppercase letters (^A,B^) indicate pairwise comparisons among ROI1, ROI2, and ROI3 for the total sample. ROIs sharing the same uppercase letter are not significantly different, whereas ROIs with different uppercase letters differ significantly. Lowercase letters (^x,y^) indicate post hoc comparisons among the Group × ROI combinations. Values sharing the same lowercase letter are not significantly different, whereas values with different lowercase letters differ significantly.

## Data Availability

The data that support the findings of this study are available from the corresponding author upon reasonable request.

## Supplementary Materials

The following supporting information can be downloaded at: https://www.mdpi.com/article/10.3390/diagnostics16162627/s1, Table S1. Pairwise comparisons between study groups. Table S2. Pairwise comparisons between ROIs. Table S3. Pairwise comparisons between study groups within each ROI.
