# Supplementary material for: Anatomical Evaluation of Localized Mandibular Trabecular Texture Differences in Children with Hypodontia Using Radiographic Fractal Analysis
Source: Diagnostics (Basel). 2026 Aug 19;16(16):2627. doi: 10.3390/diagnostics16162627 (PMC13511662; doi:10.3390/diagnostics16162627)
Supplement: Supplementary file 1 [file diagnostics-16-02627-s001.zip › diagnostics-4437538-supplementary.pdf]

## Supplementary Materials: Bonferroni-Adjusted Pairwise Comparisons Derived from the Mixed-Design Repeated-Measures ANOVA

**Table S1.** Pairwise comparisons between study groups.

| Comparison | Mean difference | 95% CI | Bonferroni-adjusted p |
|---|---|---|---|
| Group 1 vs Group 2 | 0.136 | 0.053 to 0.219 | <0.001 |
| Group 1 vs Group 3 | 0.045 | −0.038 to 0.128 | 0.557 |
| Group 2 vs Group 3 | −0.091 | −0.174 to −0.008 | 0.027 |

**Table S2.** Pairwise comparisons between ROIs.

| Comparison | Mean difference | 95% CI | Bonferroni-adjusted p |
|---|---|---|---|
| ROI1 vs ROI2 | −0.015 | −0.085 to 0.056 | 1.000 |
| ROI1 vs ROI3 | 0.072 | −0.012 to 0.156 | 0.119 |
| ROI2 vs ROI3 | 0.087 | 0.005 to 0.169 | 0.035 |

**Table S3.** Pairwise comparisons between study groups within each ROI.

| ROI | Comparison | Mean difference | 95% CI | Bonferroni-adjusted p |
|---|---|---|---|---|
| ROI1 | Group 1 vs Group 2 | 0.095 | −0.026 to 0.217 | 0.176 |
| ROI1 | Group 1 vs Group 3 | 0.112 | −0.010 to 0.234 | 0.081 |
| ROI1 | Group 2 vs Group 3 | 0.017 | −0.105 to 0.138 | 1.000 |
| ROI2 | Group 1 vs Group 2 | ~0.000 | −0.130 to 0.130 | 1.000 |
| ROI2 | Group 1 vs Group 3 | −0.039 | −0.168 to 0.091 | 1.000 |
| ROI2 | Group 2 vs Group 3 | −0.039 | −0.168 to 0.091 | 1.000 |
| ROI3 | Group 1 vs Group 2 | 0.313 | 0.150 to 0.476 | <0.001 |
| ROI3 | Group 1 vs Group 3 | 0.062 | −0.101 to 0.225 | 1.000 |
| ROI3 | Group 2 vs Group 3 | −0.251 | −0.414 to −0.088 | 0.001 |

Mean differences represent estimated marginal mean differences (I–J). Confidence intervals are 95% confidence intervals for the pairwise mean differences. P-values were adjusted for multiple comparisons using the Bonferroni method.
